# Coordinated Control of Acoustical Field of View and Flight in Three-Dimensional Space for Consecutive Capture by Echolocating Bats during Natural Foraging

**DOI:** 10.1371/journal.pone.0169995

**Published:** 2017-01-13

**Authors:** Miwa Sumiya, Emyo Fujioka, Kazuya Motoi, Masaru Kondo, Shizuko Hiryu

**Affiliations:** 1 Faculty of Life and Medical Sciences, Doshisha University, Kyotanabe, Kyoto, Japan; 2 Organization for Research Initiatives and Development, Doshisha University, Kyotanabe, Kyoto, Japan; 3 JST PRESTO, Kawaguchi, Saitama, Japan; Texas A&M University College Station, UNITED STATES

## Abstract

Echolocating bats prey upon small moving insects in the dark using sophisticated sonar techniques. The direction and directivity pattern of the ultrasound broadcast of these bats are important factors that affect their acoustical field of view, allowing us to investigate how the bats control their *acoustic attention* (pulse direction) for advanced flight maneuvers. The purpose of this study was to understand the behavioral strategies of acoustical sensing of wild Japanese house bats *Pipistrellus abramus* in three-dimensional (3D) space during consecutive capture flights. The results showed that when the bats successively captured multiple airborne insects in short time intervals (less than 1.5 s), they maintained not only the immediate prey but also the subsequent one simultaneously within the beam widths of the emitted pulses in both horizontal and vertical planes before capturing the immediate one. This suggests that echolocating bats maintain multiple prey within their acoustical field of view by a single sensing using a wide directional beam while approaching the immediate prey, instead of frequently shifting *acoustic attention* between multiple prey. We also numerically simulated the bats’ flight trajectories when approaching two prey successively to investigate the relationship between the acoustical field of view and the prey direction for effective consecutive captures. This simulation demonstrated that acoustically viewing both the immediate and the subsequent prey simultaneously increases the success rate of capturing both prey, which is considered to be one of the basic axes of efficient route planning for consecutive capture flight. The bat’s wide sonar beam can incidentally cover multiple prey while the bat forages in an area where the prey density is high. Our findings suggest that the bats then keep future targets within their acoustical field of view for effective foraging. In addition, in both the experimental results and the numerical simulations, the acoustic sensing and flights of the bats showed narrower vertical ranges than horizontal ranges. This suggests that the bats control their acoustic sensing according to different schemes in the horizontal and vertical planes according to their surroundings. These findings suggest that echolocating bats coordinate their control of the acoustical field of view and flight for consecutive captures in 3D space during natural foraging.

## Introduction

Echolocating bats have remarkable ultrasonic sensing abilities. They emit directional ultrasonic signals and listen to the echoes returning from objects. Perception by the bats firstly depends on the acoustical features of the returning echo, which change depending on various factors, such as distance to target, direction of target, and target strength. Second, the direction and directivity patterns of the beams emitted by these bats are simple but important factors that affect their “field of view” from echolocation (acoustical field of view), and restrict the extent of spatial information during echolocation. The microphone-array technique has been widely used to track the three-dimensional (3D) flight trajectory of bats [1–4] and also allows us to measure the direction and directivity of the bats’ sonar beams during flight in a flight chamber and in the field [2,4,6–13]. By measuring the pulse direction (*acoustic attention*) and directivity patterns (beam width) as useful indices, we can investigate where the bats direct their attention and potentially detect prey, and how they control their acoustical field of view during advanced flight maneuvers. Recently, it has been reported that echolocating bats actively change the pulse direction depending on the situation in a flight chamber [2,7,8,10,12] or even in the field [4,5]. They have also been reported to expand the beam width adaptively before capturing target prey to retain a moving target within the acoustical field of view [11,13] or to narrow the beam width when entering a confined space [14]. These studies demonstrated that bats actively adjust both the pulse direction and the beam width, as well as traditional acoustic characteristics, such as time-frequency structure, sound pressure level, or inter-pulse interval (IPI) [15,16].

In our previous study, we constructed a 32-ch microphone-array covering an area of 22 × 24 m, to measure the 3D flight paths and horizontal pulse directions of the Japanese house bat, *Pipistrellus abramus* during natural foraging [5]. *Pipistrellus abramus* exhibits acrobatic hunting behavior, which involves occasionally capturing multiple airborne insects within a short time interval (less than 1 s) in the field [3,5]. We also reported that the bats directed their pulse toward the subsequent target before capturing the immediate one when attacking two successive targets [5]. On the other hand, our recent study adopted a mathematical methodology to estimate parameters representing the *flight attention* of bats for their measured flight paths during the phase of approaching prey [17]. (Note that the *flight attention* is derived from a parameter of weighing factors to minimize the angular difference between the bat’s flight direction and the direction to its prey. This represents the attention by the bat toward a certain target prey in terms of flight.) We found that the distribution of the *flight attention* parameters (estimated from the behavioral data of wild *P*. *abramus* during two consecutive captures in a short-time interval) corresponded to the optimal value of the parameter set in the numerical simulation, which showed a high success rate of consecutive prey captures. This indicated that bats plan their future flight paths based on additional information about their next prey. This model was based on the assumption that bats distribute their attention across multiple prey. This implies that bats acoustically recognize the positions of multiple prey at the same time, even while approaching their immediate prey. To confirm this assumption experimentally, we investigated the relationship between the prey direction (direction of prey relative to the pulse direction) and the beam width of the bat during natural foraging in the field. We proposed two hypotheses: 1) bats physically shift their pulse direction between immediate and subsequent targets in somewhat of a time-sharing manner (time-sharing manner hypothesis), or 2) bats aim their beam across a wide area to cover both immediate and subsequent targets simultaneously within their acoustical field of view (acoustical field hypothesis). Based on our previous studies [5,17], we propose an acoustical field hypothesis in which the bats are assumed to aim their pulses in a certain direction. This keeps multiple targets within their acoustical field of view, including the peripheral part. These features may be linked to processes in visual-guided animals; for example, humans sometimes detect objects using the peripheral field of vision [18–20]. Visual-guided animals are also known to shift the gaze direction sequentially to guide movement planning [21]. Therefore, the investigation of control of the acoustical field of view by bats during flight not only provides insight into how animals actively sample spatial information from their environment during locomotion, but can also be used for comparative studies along with visual research on how animals use their vision during locomotion [7,8].

Echolocating bats seem to use their acoustical field of view effectively during aerial-feeding flights by advanced coordinated control of the acoustical field of view and flight. To investigate how bats control these features in 3D space during consecutive prey-capture flights, we established horizontal and vertical microphone-arrays in the field to measure the flight paths and 3D pulse direction and directivity pattern of the emitted sounds during natural foraging. For every pulse emission, we examined time variation in the pulse direction and beam pattern in relation to the direction of immediate and subsequent targets while attacking multiple target prey consecutively in the field. Furthermore, we conducted a numerical simulation to determine how the bats control the acoustical field of view according to the prey directions for the successful capture of both immediate and subsequent prey.

## Materials and Methods

### Subjects and study site

The subject of this study was *P*. *abramus*, which is a member of the family Vespertilionidae and has a wingspan of 10–15 cm and a bodyweight of 5–8 g. During natural foraging, *P*. *abramus* emits relatively long (9–11 ms), shallow-swept frequency-modulated (FM) pulses, with the energy concentrated in the terminal frequency of the fundamental component at around 40 kHz [3,22]. The bats are regularly observed in large open areas during the evening from early summer to fall. Here, the study site was a large open area over a river with the width of approximately 20 m, near the campus of Doshisha University in southern Kyoto Prefecture, Japan, where only *P*. *abramus* regularly appears to forage for airborne insects. Our field studies did not involve endangered or protected species. No specific permissions were required for these locations/activities because the study site was not protected by the regulatory body concerned with the protection of wildlife. The target prey of *P*. *abramus* are mainly small hemipterans and dipterans [23]. The flight speed of the common chironomid midge *Chironomus plumosus* ranges from 0.25 to 1.1 m/s [24], and we visually observed that swarming dipteran midges took several seconds to fly across an area of a few tens of centimeters. From our observation, we concluded that these prey are roughly ten times slower than *P*. *abramus* (which has an average speed of 5 m/s) and therefore assumed that the movement of the prey, at this study site, was negligible during the brief period of approach and capture (< 3 s) in each flight sequence examined in this study [17].

When a bat successfully captures an insect, a brief burst of sounds (feeding buzz) occurs, followed by a silent interval (post-buzz pause) [25–27]. Based on our previous recording, *P*. *abramus* emits a feeding buzz approximately 0.2 s before capture [3,5], at a distance of a few tens of centimeters (approximately 30 cm) from the prey point. Since the movement of prey can be negligible within this brief period of time, we simply defined the bat’s 3D position at the end of the feeding buzz as the location of the prey at the capture in this study [3,5,17].

It is difficult to draw definitive conclusions about whether a bat has successfully captured prey based only on information about the post-buzz pause [17,27]. However, we confirmed that the distances between successful prey positions were too large for the prey (small midges) to move during the observed inter-capture intervals for the flights analyzed in this study. Therefore, if the bats failed to capture prey, it is highly unlikely that they attacked the same prey again in the second capture trial just after the first unsuccessful capture. Although it is still an assumption, we assumed that the bats attacked different prey sequentially, at least in the case of two successive target captures with short time intervals (< 1.5 s) in this study. We thus categorized two successive target captures with long time intervals (> 3.0 s) as *long-interval capture* and those with short time intervals (< 1.5 s) as *short-interval capture*.

### Large-scale 3D microphone-array system

The recordings were carried out on 5 separate days (October 14, 2013; July 15, September 30, October 7, and October 16, 2014) for approximately one hour before and after sunset. Recording was conducted for 5–10 min each session and the total number of recording sessions was 32 for 5 days (total recording time: 225 min). To reconstruct the flight paths of the bats and identify the capture points accurately, we selected prey-capture flights where the amplitude of the sonar sounds was sufficiently high with a good signal-to-noise ratio, even at the end of the terminal buzz. At the same time, to ensure measurement accuracy, we selected only flights where the bat approached the targets while flying toward the L-shaped array inside of the U-shaped array. Thus, the vertical and horizontal pulse directions and beam widths could be measured accurately. We analyzed a total of 2,680 pulses from 37 captures in 20 measured flight paths. That is, flight paths with three successive captures were split into two flight paths with two successive captures each. (See Results.)

**Fig 1:**
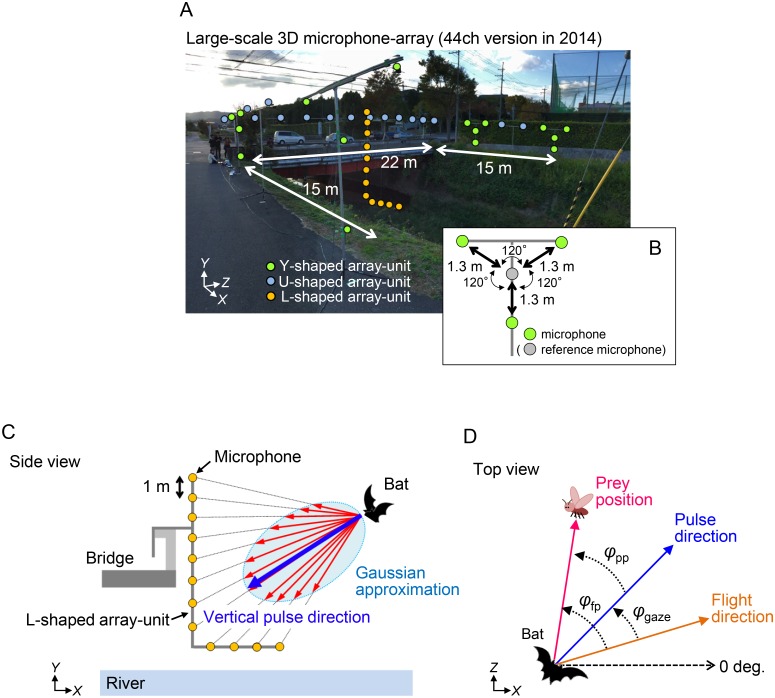
Large-scale 3D microphone-array system. (A) Photograph of study site and microphone-array system with 44 microphones consisting of U-shaped 32-ch microphone-array and L-shaped 12-ch microphone-array in 2014. Four Y-shaped arrays (green dots) are part of the U-shaped array. Total of 24 microphones distributed over the entire U-shaped array at the same horizontal level were used to measure horizontal pulse direction, whereas L-shaped array units (orange dots) measured vertical pulse direction. Y-shaped array was used to reconstruct 3D flight paths of the bats. (B) A schematic diagram of the Y-shaped array unit. (C) Side view of the L-shaped array unit. The vertical pulse direction (blue arrow) was determined from the peak of a Gaussian curve (light blue curve), based on the sound pressure vectors (red arrows) across all 12 microphones. The horizontal pulse direction was also determined by the same procedure using the horizontal U-shaped microphone-array. (D) Definitions of the positional relationship between the bat and the target. The gaze angle *φ*_gaze_ (or *θ*_gaze_) was the pulse direction (blue arrow) relative to the flight direction (yellow arrow) of the bat. The directions of the capture positions (prey position) *φ*_fp_ (or *θ*_fp_) and *φ*_pp_ (or *θ*_pp_) were the prey direction (magenta arrow) relative to the flight direction and the pulse direction of the bat, respectively. Here, *φ* is the horizontal angle and *θ* is the vertical angle. (See S1 Table).

Echolocation pulses emitted from the bats were recorded using a custom-built 44-ch 3D microphone-array (Fig 1A). We previously reconstructed the horizontal pulse direction along with 3D flight paths using a horizontal U-shaped 32-ch microphone-array system [5]. In this study, we newly built a vertical 12-ch L-shaped microphone-array unit so that the vertical pulse direction could be measured. The data shown here are from measurements using two different configurations with 32 microphones in 2013 (vertical L-shaped 10-ch and horizontal U-shaped 22-ch), and 44 microphones in 2014 (vertical L-shaped 12-ch and horizontal U-shaped 32-ch).

The microphone-array units were arranged to cover the entire foraging area over the stream, as shown in Fig 1A. We reconstructed the 3D flight paths using four Y-shaped array units using omnidirectional electret condenser microphones (models FG-23329-C05 and FG-23629-P16; Knowles, Itasca, IL, USA). The emitted echolocation pulses were recorded and amplified using a custom-built electronic circuit via a 10–250 kHz band-pass filter, and were digitized with 16-bit accuracy at a sampling rate of 500 kHz using high-speed data acquisition cards (PXIe-6358; National Instruments, Tokyo, Japan). The output signals were synchronously stored using a personal computer via a custom program using LabVIEW 2011 (National Instruments).

### Reconstruction of 3D flight paths and pulse directions

The 3D locations of the bats were obtained using the difference in arrival times between a central and three other microphones separated by 1.3 ± 0.01 m in the Y-shaped array (Fig 1B). The arrival-time differences were calculated from cross-correlation functions using a Matlab routine (Math Works, Natick, MA, USA), and the analytical procedure was the same as in our previous studies [3,5,17]. We combined 3D sound coordinates calculated by each of four Y-shaped array units so that the flight trajectory could be reconstructed within the foraging area, which was enclosed by the microphone-arrays. The maximum range error of the microphone-array system was less than 10 cm for sound sources within 5 m of the Y-shaped array unit [3].

The horizontal pulse direction was calculated based on the sound pressure difference, namely, the differences in peak power in the spectrogram of a sonar sound across 24 microphones that were distributed over the entire array at the same horizontal level. The vertical pulse direction was also measured by the L-shaped array unit by the same method (Fig 1C). The sound pressure levels of the pulses were corrected for the propagation loss of sounds in the air between the bat and each microphone and the sensitivity differences between the microphones in the array. Sound absorption was calculated from measured absorption coefficients that were determined for the average frequencies at the peak energy in the FM pulse of *P*. *abramus* (1.2 dB/m at 45 kHz). The sensitivity of the microphone-array elements was measured by sending a 10-ms burst at 45 kHz to each microphone of the array using an ultrasonic loudspeaker (PT-R7, Pioneer, Tokyo, Japan). This allowed the recorded sounds to be calibrated according to sensitivity differences between the microphones. The directivity pattern of the sonar beam was reconstructed by curve fitting using a Gaussian function, and the direction at the peak value of the reconstructed directivity pattern was determined as the pulse direction. The beam width was defined by –6 dB off-axis angles in the reconstructed directivity pattern from the pulse direction. In our previous studies, we calibrated the measurement accuracy of the pulse direction and beam width for a U-shaped array. The error of the pulse direction was less than ±5°, and that of the beam width was less than ±7°, in the horizontal plane when the sonar beam was directed toward the U-shaped horizontal microphone-array [5,17]. In the present study, prior to the experiments, we arranged the L-shaped arrays in the field following the actual recording setup to measure the error of the vertical pulse direction and the beam width by using artificial FM sounds emitted from a loudspeaker (PT-R7; Pioneer, Tokyo, Japan). As a result, when the bat’s sonar beam was directed to the inside of the L-shaped array (as shown in Fig 1C), the errors for both the vertical pulse direction and the beam width were less than ±5°. The amount of error in this study corresponded to that of our previous studies [5,17], which was an acceptable amount of error to investigate the acoustical behavior of bats at the study site. To ensure measurement accuracy, we measured the horizontal beam width only when the vertical pulse direction was within ±45° from the horizontal plane of the U-shaped horizontal microphone-array. Therefore, when the bats emitted a pulse toward the exterior of the microphone-array (i.e., the positive direction in the *X*-axis in Fig 1A), we did not use the data for calculating beam width, but rather used only that for pulse direction to ensure the measurement accuracy of the beam width because it was unclear whether the vertical pulse direction was directed within ±45° from the horizontal plane. The vertical pulse direction was reconstructed only when the horizontal pulse direction was toward the inside of the U-shaped microphone-array. The vertical beam width was also calculated only when the horizontal pulses were directed within ±45° from negative direction of *X*-axis (Fig 1A) to ensure measurement accuracy.

In a strict sense, because auditory perception depends on the level of the returning echo and the hearing threshold of the bat [28], we should consider not only the beam width but also the angular range, combined with the transfer function of the outer ear, to define the acoustical field of view [29,30]. In this study, however, we adopted the −6 dB point of the beam width as a simple index of the acoustical field of view during echolocation following the approach used in recent studies [31,32].

We defined the positional relationship between the bat and the prey position, as shown in Fig 1D. The variables *φ* and *θ* represent the horizontal and vertical angles, respectively. The bat’s horizontal (vertical) gaze angle *φ*_gaze_ (*θ*_gaze_) was defined as the pulse direction relative to the flight direction. When the bat captured multiple successive targets, we used suffixes to represent the order of the captures. *φ*_fp_ (or *θ*_fp_) was defined as the direction of the prey position relative to the flight direction of the bat, while *φ*_pp_ (or *θ*_pp_) indicates the direction of the prey position relative to the pulse direction. (See S1 Table.)

### Acoustic analysis

The analytical procedure was also the same as that used in our previous studies [3,5]. The acoustic characteristics, including IPI and pulse duration, were analyzed from echolocation sounds recorded by the central microphone of the Y-shaped array unit that was spatially closest to the sound source (the bat) and thus received the strongest version of each broadcast using an additional custom program in Matlab. IPI was determined as the time between the amplitude envelope peaks of successively emitted echolocation pulses on oscillograms. The pulse duration was determined from a spectrogram of the first harmonic component of an extracted individual pulse (1024 points fast Fourier transform with a Hanning window, 97% overlap) at −25 dB relative to the peak intensity of the pulse. The foraging behavior of the bats was categorized into three phases based on the characteristics of the sonar sound; namely, a search phase, an approach phase, and a terminal phase [33]. The terminal phase of pipistrelles is usually divided into two signals: buzz I and buzz II [26]. Buzz II signals have lower bandwidth and frequency than buzz I signals [33]. Since the start of the approach phase of wild *P*. *abramus* was characterized by the appearance of a slight increase in the pulse duration [3,5], we used this as a simple index for identifying the timing of the beginning of the approach phase in this study.

For statistical comparisons, a *t*–test and Mardia–Watson–Wheeler test were used, when appropriate, to test for significant differences in beam width and angular variables between data sets.

### Methods for the numerical simulation

We numerically simulated the flight trajectory of a bat when approaching two prey successively, so that the relationships between the acoustical field of view and the direction of each prey could be investigated quantitatively. In this framework, the bat dynamically changes its flight direction depending on the directions of the two prey, using the mathematical model proposed in our previous study [17]. Briefly, the modeled flight dynamics in the horizontal and vertical planes can be described as follows:

$$\frac{d\varphi_{\text{b}}(t)}{dt}=\frac{1}{\delta_{\text{h}}}\{\alpha_{\text{h}}\text{sin}[\varphi_{\text{bp}1}(t)-\varphi_{\text{b}}(t)]+\beta_{\text{h}}\text{sin}[\varphi_{\text{bp}2}(t)-\varphi_{\text{b}}(t)]\},$$ (1)

$$\frac{d\theta_{\text{b}}(t)}{dt}=\frac{1}{\delta_{\text{v}}}\{\alpha_{\text{v}}\text{sin}[\theta_{\text{bp}1}(t)-\theta_{\text{b}}(t)]+\beta_{\text{v}}\text{sin}[\theta_{\text{bp}2}(t)-\theta_{\text{b}}(t)]\},$$ (2)

where *δ* represents positive weighting factors and *α* is the minimization of the angular difference between the bat’s own flight direction [*φ*_b_(*t*), *θ*_b_(*t*)] and the direction to prey 1 [*φ*_bp1_(*t*), *θ*_bp1_(*t*)] (similar for *β* to prey 2). To simplify the numerical simulation, we constrain the parameters as follows:

$$\alpha_{\text{h}}{}^{2}+\beta_{\text{h}}{}^{2}=1,$$ (3)

$$\alpha_{\text{v}}{}^{2}+\beta_{\text{v}}{}^{2}=1.$$ (4)

Therefore, parameters *α*_h_ and *β*_h_ (*α*_v_ and *β*_v_) are described as *α*_h_ = sin*γ*_h_ and *β*_h_ = cos*γ*_h_ (*α*_v_ = sin*γ*_v_ and *β*_v_ = cos*γ*_v_). To examine the ratio of the *flight attention* between the two prey, the arctangent of parameters *α* and *β* is defined by *γ*: namely, *γ*_h_ and *γ*_v_ represent the arctangents in the horizontal and vertical planes, respectively.

The flight path was numerically simulated for a situation in which two prey are distributed inside the bat’s 3D sonar beam, which was modeled as a circular piston oscillating in an infinite baffle [11]. A simulation trial began when the bat started its approach phase to capture prey 1. The calculation conditions for a parameter set (*γ*_h_ and *γ*_v_) are the same as those in our previous study [17], and the beam widths of the sonar beam in the horizontal and vertical planes were defined as ±59° and ±25°, respectively, which were derived from the experimental results of this study. Three trials were performed for each parameter set of *γ*_h_ and *γ*_v_, ranging from −π to π, respectively, with 0.01π steps, and 201 × 201 pairs. All variables in this model were calculated by using the fourth-order Runge–Kutta method, with a time step of 0.01 s (the flight speed of the model bat was taken to be 5 m/s based on the experimental data). Pulses to obtain the target positions were emitted at every step. We examined the relationships between the acoustical field of view and the direction of each prey, in cases of both success and failure of the prey-capture trials, using this mathematical model. A parameter set was defined as a success when the bat captured (close in within 10 cm) the immediate prey (prey 1) and then the subsequent prey (prey 2) in sequence (capturing both prey), without losing the location of the prey which the bat intend to capture (i.e., prey 1 before the capture of prey 1 and prey 2 after the capture of prey 1). A simulation trial was defined as a failure when prey 2 was located outside the sonar beam after the capture of prey 1 (capturing only prey 1) (note that cases in which both prey 1 and prey 2 were missed were not considered in the investigation). We categorized the numerical simulation results of all trials into cases of success and failure and then analyzed the relationships between the acoustical field of view and prey directions while approaching prey 1 and prey 2 sequentially. Initial positions of prey 1 and prey 2 were randomly determined in horizontal and vertical space in every trial.

## Results

### 3D flight paths and pulse directions of bats attacking multiple targets in the field

**Fig 2:**
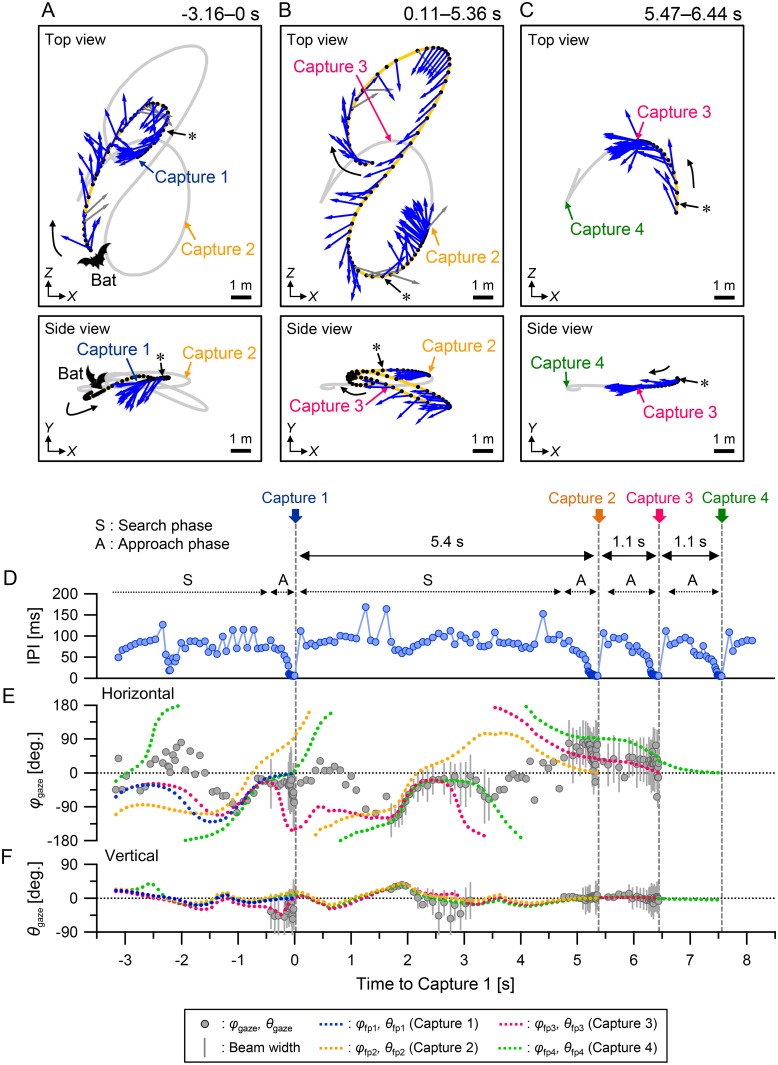
Typical example of multiple consecutive capture flight of *Pipistrellus abramus* in the field. (A–C) Top (top panels) and side (bottom panels) views of the 3D flight path and pulse directions of the bat attacking four successive targets. The observed 3D flight path of the bat was separated into three sections according to the timing of each consecutive target capture; namely, Captures 1–2 (A), Captures 2–3 (B), and Captures 3–4 (C). The black curved arrows indicate the initial flight direction of the bat. The blue arrows indicate the directions of pulse emission by the bat. The asterisks show the position where the bat started the approach phase. The gray arrows indicate the pulse emitted toward the out of the U-shaped microphone-array in the horizontal plane. Only pulses emitted before the bat captures its immediate prey are shown in the figure. (D–F) Time series data of IPIs (D), gaze angles (*φ*_gaze_ and *θ*_gaze_), and directions of prey positions in the horizontal (E) and vertical planes (F) during this flight. The beam width of the sonar beam is equivalent to the length of gray vertical lines on the gray plots. *φ*_fp_ (or *θ*_fp_) indicates the direction of the prey position relative to the flight direction of the bat. (See S1 Table.)

Fig 2A–2C show representative data of the 3D flight paths with pulse directions of *P*. *abramus* when the bat captured four insects (Targets 1–4) consecutively in 12 s. The pulse directions did not always coincide with the flight direction, especially in the horizontal plane. The bat changed its flight direction and pulse direction dynamically on either the right or the left side from its flight direction or shifted between different directions. On the other hand, 82% of all pulses whose vertical pulse direction could be measured in this flight case (shown as a blue arrow in Fig 2A–2C) were emitted downward (< 0°), whereas only 18% of the pulses were emitted upward (≥ 0°). This suggests that the bat emitted most of its pulses downward from the horizontal plane in the vertical plane.

Fig 2D shows time series data of IPI during the flight shown in Fig 2A–2C. When the bat started the approach to capture an insect, the IPI was decreased from approximately 100 to 5 ms. In this flight case, the time intervals between successive captures (Captures 2–3 and 3–4) were both 1.1 s, whereas the time interval for the first two successive captures (Captures 1–2) was 5.4 s. Throughout this flight period, the horizontal gaze angle, *φ*_gaze_, was widely distributed from approximately −130° to 80° (Fig 2E). In contrast, in the vertical plane, the gaze angle *θ*_gaze_ had a narrower range from about −60° to about 40° (Fig 2F), indicating that the bat changed the horizontal acoustical field of view more dynamically than the vertical one during foraging.

Fig 2E and 2F show that *φ*_gaze_ and *θ*_gaze_ often corresponded to the directions of some of the subsequent prey positions; that is, not only the prey position of the immediate target but also subsequent target prey positions corresponded to the center of the acoustical field of view. In addition, both the immediate and subsequent prey positions were simultaneously within the −6 dB beam width of the sonar beam (gray vertical lines). In particular, Fig 2E shows that the pulse direction corresponded to the positions of Captures 3 and 4, approximately 4 s before Capture 2 occurred. (See from 1.5 s to 3.5 s on the horizontal axis.)

### Future prey positions were covered by acoustical field of view

**Fig 3:**
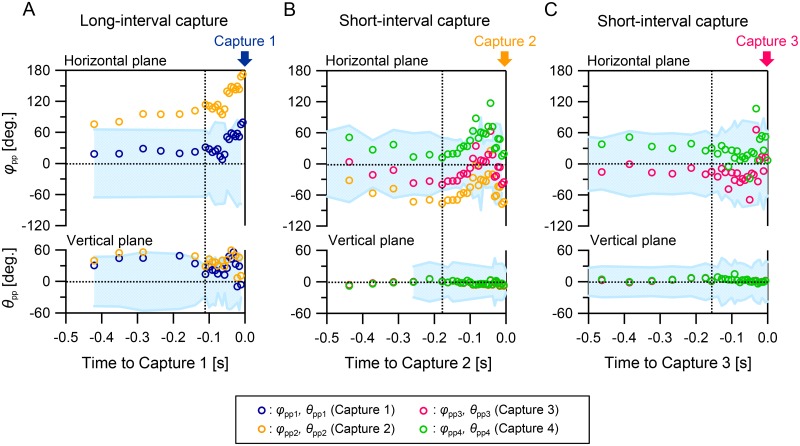
Relationship between target positions and bat sonar beam while approaching targets. (A–C) Time series data of *φ*_pp_ (top panels) and *θ*_pp_ (bottom panels) during the approach and terminal phases in *long-* (A) and *short-interval captures* (B, C) shown in Fig 2A–2C. *φ*_pp_ (or *θ*_pp_) indicates the direction of the prey position relative to the pulse direction. (See S1 Table.) The light blue areas show the range of the −6 dB beam width of the bat’s sonar beam relative to the bat’s pulse direction. The vertical dashed lines show the timing of transition from the approach phase to the terminal phase.

Fig 3A–3C show time series data of target directions before capture of the immediate target (0.5 s) and we could define the capture points of subsequent targets as the positions of the prey themselves in the case of *short-interval capture*. Fig 3A shows that in the case of *long-interval capture*, the subsequent prey (*φ*_pp2_ and *θ*_pp2_) were outside the acoustical field of view during the approach period of the immediate target. This suggests that the bat acoustically focused only on the immediate prey during *long-interval capture*. On the other hand, during *short-interval capture*, the subsequent target prey positions were within the beam width of the bat’s emissions during the whole or part of this period in both horizontal and vertical planes (Fig 3B and 3C). These results suggest that the bats can detect the positions of both their immediate prey and subsequent ones at the same time by keeping them within their acoustical field of view before capturing the immediate one during a *short-interval capture*. Fig 3A also shows that the immediate target directions (*φ*_pp1_ and *θ*_pp1_) were not always maintained at the center of the acoustical field of view, but were within the bat’s −6 dB beam width, in both horizontal and vertical planes, in the case of *long-interval capture*.

**Fig 4:**
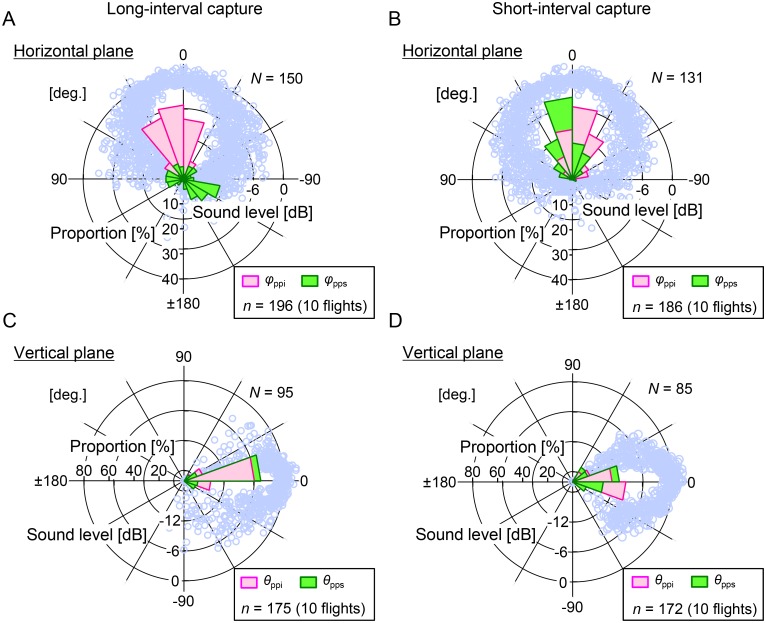
Relationship between the prey direction relative to pulse direction and the directivity patterns of the sonar beam as bats converge on immediate prey in *short-* and *long-interval captures*. Circular histograms show the directions of the immediate (horizontal, *φ*_ppi_; vertical, *θ*_ppi_) and subsequent (horizontal, *φ*_pps_; vertical, *θ*_pps_) prey relative to the bat’s pulse direction. Data were obtained from the recording sounds while the bat converged immediate prey (except for buzz II) for 10 flights each of (A, C) *long-* and (B, D) *short-interval captures*. The open light-blue circles indicate the amplitude measured at each microphone, showing the directivity patterns of the sonar beam. We defined 0 dB as the peak value of the curve-fitted directivity pattern of each sound. The vertical (A, B) and horizontal (C, D) axes for the circular histogram show the proportion relative to each number of pulses. Note that the sample sizes in the circular histogram differ from those in the directivity patterns. This is because the beam width data were analyzed only for pulses whose horizontal and vertical pulse directions could be appropriately measured at the same time. (See Materials and Methods).

Fig 4A–4D show the relationship between the target direction and −6 dB beam width during the approach phase and buzz I in terminal phase for all recorded flight data (20 flights). The time of the measured flight paths ranged from 2 to 18 s, with an average of 7.5 s. In the *long-interval captures* (10 flights), the horizontal and vertical mean −6 dB beam widths of the pulses were ±51 ± 13° (*N* = 150 pulses) and ±27 ± 14° (*N* = 95 pulses), respectively. On the other hand, in the *short-interval captures* (10 flights), the horizontal and vertical mean −6 dB beam widths of the pulses were ±59 ± 12° (*N* = 131 pulses) and ±25 ± 8° (*N* = 85 pulses), respectively. Namely, the horizontal mean beam widths were significantly wider than the vertical ones for both *short*- (the *t*–test, *t*(214) = 25.1, *P* < 0.001) and *long-interval capture* cases (the *t*–test, *t*(195) = 13.7, *P* < 0.001). In addition, the *short-interval capture* case showed a slightly but significantly wider horizontal beam width than the *long-interval capture* case (the *t*–test, *t*(279) = 5.2, *P* < 0.001), whereas the vertical ones were not significantly different between *short*- and *long*-*interval capture* cases (the *t*–test, *t*(153) = 1.06, *P* = 0.146).

Fig 4A–4D also show the directions of the immediate and subsequent prey relative to the bat’s pulse direction accompanying beam patterns while approaching the immediate prey. We found that in the cases of *long-interval captures*, only 30% (59/196 pulses) of pulses covered the subsequent prey within the mean beam width (i.e., ±51 ± 13°), whereas the immediate prey was covered by 98% (193/196 pulses) of pulses in the horizontal plane (Fig 4A). On the other hand, most of the pulses emitted while approaching the immediate prey in the case of *short-interval captures* (90%, 167/186 pulses) covered the directions of both immediate and subsequent prey within the mean beam width in the horizontal plane (i.e., ±59 ± 12°) (Fig 4B). In the vertical plane, the immediate and subsequent prey directions, *θ*_ppi_ and *θ*_pps_, were covered within the vertical mean beam width (i.e., ±27 ± 14°) by 94% (164/175 pulses) and 93% (162/175 pulses) of pulses, respectively for *long-interval captures* (Fig 4C). In the case of *short-interval captures* (Fig 4D), 81% (140/172 pulses) of pulses in the subsequent prey direction, *θ*_pcs_, were observed within the vertical mean beam width (i.e., ±25° ± 8°). The horizontal mean directions of the subsequent prey *φ*_pps_ in the cases of *long-* and *short-interval captures* were −106° (*long-interval captures*) and 9° (*short-interval captures*), respectively, which were significantly different (the Mardia–Watson–Wheeler test, *B*(2) = 165.1, *P* < 0.001). In addition, those in the vertical plane were also significantly different (the Mardia–Watson–Wheeler test, *B*(2) = 32.1, *P* < 0.001), whereas those were 8° and 7°, respectively. This shows that the bats maintained the subsequent prey within their 3D acoustical field of view in the *short-interval captures*; this supports the acoustical field hypothesis.

**Fig 5:**
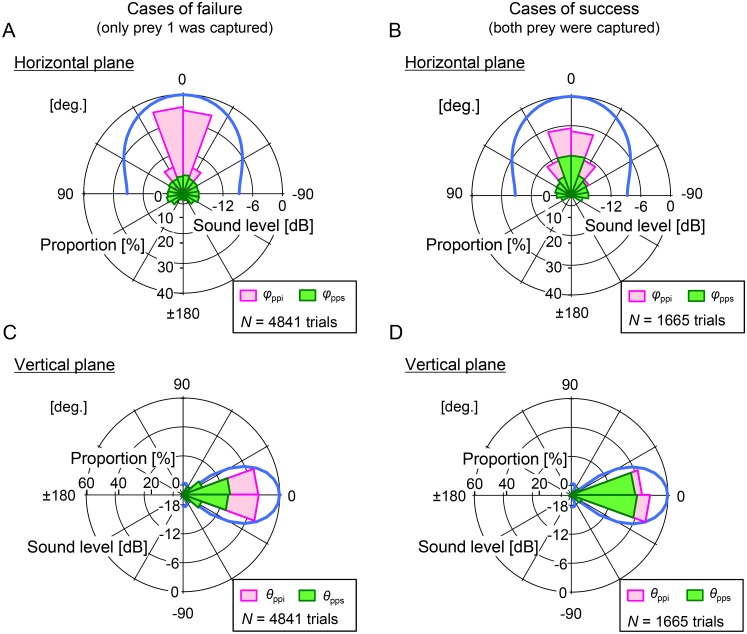
Numerical simulation of relationship between prey direction relative to pulse direction and directivity patterns of sonar beam as bats converge on immediate prey during failure and success cases. Circular histograms show the directions of the immediate (horizontal, *φ*_ppi_; vertical, *θ*_ppi_) and subsequent (horizontal, *φ*_pps_; vertical, *θ*_pps_) prey relative to the bat’s pulse direction calculated based on the simulation results. A simulation starts when the bats start to converge on immediate prey, and pulses to obtain the target positions were emitted at every step. A parameter set was defined as a success when the bat captured (close in within 10 cm) the immediate prey (prey 1) and then the subsequent prey (prey 2) in sequence (capturing both prey), without losing the location of the prey which the bat intend to capture. The simulation was assumed for the phase in which the bat converge immediate prey. Data were taken from 4,841 trials of failure (A, C) and 1,665 trials of success cases (B, D). The light blue lines show the directivity patterns of the sonar beam used in the numerical simulation.

Simultaneous coverage of both immediate and subsequent prey within the acoustical field of view is supposed to be suitable for planning of the bats’ future path to ensure capture of both prey. To test this, we conducted a numerical simulation (Fig 5). The numbers of simulation trials of failure and success were and 4,841 (229,706 pulses) and 1,665 (68,466 pulses), respectively. In the case of failure (only prey 1 was captured), only 39% (89,323/229,706 pulses from 4,841 trials) of pulses covered the subsequent prey within the beam width in the horizontal plane (Fig 5A, *φ*_pps_). On the other hand, in the success cases, 62% (42,418/68,466 pulses from 1,665 trials) of pulses covered the subsequent prey (prey 2) within the beam width before the capture of the immediate prey (prey 1) in the horizontal plane (Fig 5B, *φ*_pps_). This also suggests that the bat could potentially succeed in capturing both prey without invariably keeping the subsequent prey within the beam width in the horizontal plane; that is, the bat could occasionally lose the location of prey 2 just after the start of the simulation trial but could find it while changing the flight direction to capture the prey 1 because the pulse direction was equal to flight direction in the numerical simulation. In the vertical plane, 66% (151,655/229,706 pulses) of pulses in the failure cases and 87% (59,253/68,466 pulses) of pulses in the success cases covered both prey 1 and prey 2 (Fig 5C and 5D). These results suggest that acoustically viewing both the immediate and the subsequent prey simultaneously increases the success rate for the bats to capture both prey.

## Discussion

### Relationship between acoustic sensing and bat flight

In this study, we found that, in the case of *short-interval capture*, the bats simultaneously maintained both their immediate and subsequent prey within the horizontal and vertical beam width of the emitted pulse. In addition, the numerical simulations in this study demonstrated that keeping both targets within the beam width increased the success rate of consecutive prey captures. This is considered to be a basic axis of efficient route planning for consecutive-capture flights. These findings suggest that the bats control their pulse direction to cover multiple targets simultaneously within their acoustical field of view. This supports the acoustical field hypothesis derived from our previous studies [5,17].

One of our previous studies showed that, in the case of capturing multiple prey within a short time interval, the sonar beam of *P*. *abramus* during foraging shifted directions predictably between the current target and the next target [5]. Based on the measurement results for the 3D beam width in the present study, we found that the bats actually encompassed both prey in their acoustical field of view. This suggested that when attacking two successive targets, the bats can focus their *acoustic attention* on a subsequent target before capturing the immediate one. This observed acoustic behavior can account for the flight dynamics for the *flight attention* of bats. That is, the bat distributes its *flight attention* between the immediate and subsequent prey so that it can plan its future flight path for a high success rate of consecutive prey captures [17].

Interestingly, our experimental data show that the bats did not always maintain their immediate prey at the center of the beam width (e.g., Fig 3A and 3B). Instead, they kept the targets within the beam width including the peripheral part, using their wide directional beam effectively to distribute their *acoustic attention* among multiple targets. Such practical operation of wide directional beam scanning employed by the bats is different from the design concepts of existing sensing methods, namely, ultrasonography and radar, which employ high-speed spatial scanning of a narrow directional beam to maintain the spatial resolution of echoes.

Because the movement speed of the prey was insignificant when compared to that of the bats during aerial-feeding flights, at least for a brief period (< 3 s), we set the capture point as the target position during the approach and terminal phases. (See Materials and Methods.) On the other hand, we often see the target prey of *P*. *abramus* (i.e., mainly small hemipterans and dipterans) [23] swarming at the same position for several tens of seconds, implying that *P*. *abramus* in our study site may use information on patch locations of insect swarming for consecutive captures. For example, Fig 2E shows that the pulse direction corresponded to the positions of Captures 3 and 4, approximately 4 s before Capture 2 occurred. (See from 1.5 s to 3.5 s, in the horizontal axis.) It is still difficult to conclude, however, that the coincidence of the pulse direction with the future prey positions implies that the bat might obtain information on the subsequent prey in advance beyond our expectations. There is another possibility: other prey might be incidentally covered by the acoustic beam before the bat captures its immediate prey during flight. That is, the bat’s sonar beam could incidentally cover multiple prey while it forages in an area where the prey density is high. Therefore, at the beginning of approaching the consecutive prey, this would be less of a strategy than a coincidence with respect to keeping multiple prey within the bat’s acoustical field of view.

Our previous numerical simulation, however, revealed that a bat’s active utilization of positional information on subsequent prey is effective for planning future flight paths during *short-interval captures* [17]. Furthermore, the modeling results in the present study support the idea that maintaining multiple targets within one sonar beam is an effective strategy for consecutive capture. Based on all of these findings, we suggest that the bat’s wide sonar beam incidentally covers multiple prey, and then the bat keeps future targets within its acoustical field of view by actively controlling its acoustical field of view for effective foraging. Mathematical modeling allows us to analyze animal behavior quantitatively, while also suggesting new insights and implementations of cause and effect [e.g., 34, 35]. Further experimental and mathematical investigations into how capable bats are at prediction, and the utilization of the gathered information, will be helpful to clarify the collaborative control of the acoustical field of view and flight path planning employed by bats, in the context of effective adaptation of foraging behavior.

### Acoustic sensing in the horizontal and vertical planes

The experimental results demonstrated that the scanning behavior of *P*. *abramus* differed in the horizontal and vertical planes. We found that the vertical scanning range and the sonar beams were narrower than the horizontal ones in both *long-* and *short-interval capture* flights. (See Fig 4A–4D.) The bat changed the horizontal acoustical field of view more dynamically than the vertical one, sampling spatial information preferentially in the horizontal plane. In addition, the vertical flight range of the bats (variation of flight height) was also narrower by over five times than the horizontal one. (See Fig 2A–2C.) Namely, the bats modify their *flight attention* on the horizontal plane for prey that are incidentally on the same vertical plane. Prey insects are usually limited to a certain height above the water. The bats at our study site must avoid bumping into the riverbank during their acrobatic flight maneuvers. Hence, the physical restrictions of the study site may dictate that bats mostly scan their environment horizontally.

On the other hand, the experimental results show that the acoustical coverage rate of subsequent prey in the vertical plane (93% of pulses, Fig 4C) was higher than that in the horizontal plane in the case of *long-interval capture* (30%, Fig 4A), which is consistent with the numerical simulations of failure cases (capturing only prey 1) (Fig 5A vs. 5C). Experimental and mathematical results suggest that the bats tend to search for target prey distributed within a certain altitude range, which results to narrow down the vertical scanning and flight ranges. Foraging *P*. *abramus* often emits pulses downward (see Results) and attacks insect prey in descending flights [3]. Simplifying sensing and flight control in the vertical plane may be effective for complex aerial-feeding flight in 3D space.

## Supporting Information

S1 Table Definitions of angular variables. (TIF)
