# Supplementary figures and images for: Coordinated Control of Acoustical Field of View and Flight in Three-Dimensional Space for Consecutive Capture by Echolocating Bats during Natural Foraging
Source: PLoS One. 2017 Jan 13;12(1):e0169995. doi: 10.1371/journal.pone.0169995 (PMC5234808; doi:10.1371/journal.pone.0169995)

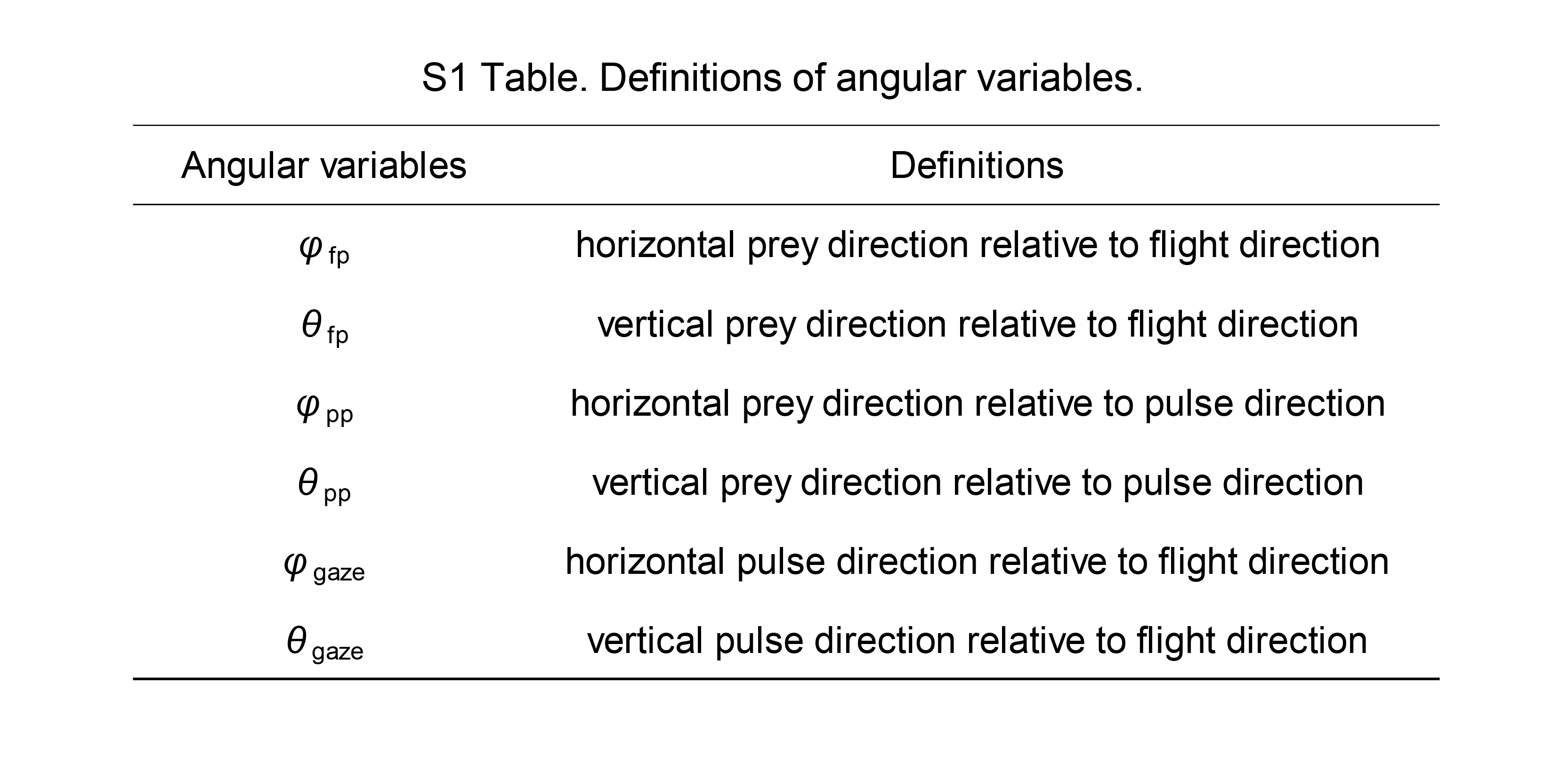

Supplement: S1 Table — (TIF) [file pone.0169995.s001.tif]
